# Anti-Biofilms’ Activity of Garlic and Thyme Essential Oils against *Salmonella typhimurium*

**DOI:** 10.3390/molecules27072182

**Published:** 2022-03-28

**Authors:** Alaa Eldin M. A. Morshdy, Ahmed S. El-tahlawy, Sameer H. Qari, Alaa T. Qumsani, Daniyah Habiballah Bay, Rokayya Sami, Eman Hillal Althubaiti, Ahmed M. A. Mansour, Amani H. Aljahani, Abd El-Salam E. Hafez, Abdallah Fikry A. Mahmoud, Rasha M. El Bayomi, Mohamed A. Hussein

**Affiliations:** 1 Food Control Department, Faculty of Veterinary Medicine, Zagazig University, Zagazig 44519, Egypt; ammorshdy@vet.zu.edu.eg (A.E.M.A.M.); aahafez@vet.zu.edu.eg (A.E.-S.E.H.); afmahmoud@vet.zu.edu.eg (A.F.A.M.); rmazab@zu.edu.eg (R.M.E.B.); elged@zu.edu.eg (M.A.H.); 2 Department of Biology, Al-Jumum University College, Umm Al-Qura University, Makkah 21955, Saudi Arabia; shqari@uqu.edu.sa (S.H.Q.); atqumsani@uqu.edu.sa (A.T.Q.); 3 Department of Biology, Faculty of Science, Umm Al-Qura University, Makkah 21955, Saudi Arabia; dhgarybay@uqu.edu.sa; 4 Department of Food Science and Nutrition, College of Sciences, Taif University, P.O. Box 11099, Taif 21944, Saudi Arabia; 5 Department of Biotechnology, Faculty of Science, Taif University, P.O. Box 11099, Taif 21944, Saudi Arabia; i.althubaiti@tu.edu.sa (E.H.A.); ahmed_amin64@yahoo.com (A.M.A.M.); 6 Department of Physical Sport Science, College of Education, Princess Nourah bint Abdulrahman University, P.O. Box 84428, Riyadh 11671, Saudi Arabia; ahaljahani@pnu.edu.sa

**Keywords:** essential oils, *S. typhimurium*, *SdiA* gene, antibiotic, biofilm formation

## Abstract

Biofilm control by essential oil (EO) application has recently increased to preclude biofilm production on foods and environmental surfaces. In this work, the anti-biofilm effects of garlic and thyme essential oils using the minimum inhibitory concentration (MIC) method against *Salmonella typhimurium* recovered from different abattoir samples were investigated along with the virulence genes (*InvA, SdiA* and *Stn* genes), and the antimicrobial susceptibility profile of *S*. *typhimurium* as well. The obtained results revealed that *S*. *typhimurium* contaminated abattoir samples to varying degrees. The *InvA* gene was investigated in all isolates, whereas the *SdiA* and *Stn* genes were observed in four and three isolates, respectively. Utilizing the disc diffusion method, *S*. *typhimurium* isolates demonstrated substantial resistance to most of the examined antibiotics with a high multiple antibiotic resistance index. *S*. *typhimurium* isolates demonstrated biofilm formation abilities to various degrees at varied temperatures levels (4 °C and 37 °C). In conclusion, the obtained samples from the research area are regarded as a potential *S*. *typhimurium* contamination source. Furthermore, garlic essential oil (GEO) has more potential to inhibit *S*. *typhimurium* biofilm at different sub-minimum inhibitory concentrations as compared to thyme essential oil (TEO). Therefore, these EOs are considered as potential natural antibacterial options that could be applied in food industry.

## 1. Introduction

*Salmonella* is considered as one of the most pervasive sources of bacterial gastroenteritis in human beings worldwide. Because beef carcasses in slaughterhouses are commonly contaminated with the species of *Salmonella*, so they could be significant sources of the pathogen in humans [1]. *Salmonella* serotypes are significantly present in nature and also found in the intestines of all animal species, resulting in a wide range of *Salmonella* infection sources [2]. Furthermore, contaminated floors, walls, and abattoir effluents are considered a major source of *Salmonella* species contamination [3,4]. *Salmonella* serotypes such as *Salmonella typhimurium* are hazardous because they cause food poisoning and develop biofilms that are difficult to remove during the cleaning process [5]. Antibiotic resistance in biofilm-producing bacteria is occasionally 1000 times higher than in planktonic ones [6].

Biofilms can be defined as multicellular matrices and a predominant approach that bacteria use to adhere to several external environments [7,8]. It has been discovered that the biofilm cells resistance is multifactorial and is linked to the barrier results from the extracellular polymeric constituents, the physiological heterogeneity, and resistance genes expression [9,10]. *S. typhimurium* strains have different abilities to develop divergent biofilms on surfaces frequently found in food environments [11,12]. Furthermore, several studies have established that the capability of *S. typhimurium* to produce biofilms on different surfaces enhances its persistence in different environments by increasing its physical and chemical resistance [13,14]. Accordingly, understanding the aspects of *S*. *typhimurium* biofilm formation is critical for developing microbial control strategies.

The primary reasons for the rise in bacterial disease prevalence are the shortage of adequate therapeutic approaches, multi-resistance to antibiotics, and the tendency of bacteria to produce biofilm [15]. Consequently, novel strategies for mitigating the resistance of *Salmonella* as a high-incidence pathogen and regulating bacterial biofilms have recently emerged, emphasizing cutting-edge technology such as natural antimicrobials. Essential oils (EOs) are considered antimicrobial agents obtained from herbal plants utilizing numerous extraction approaches. Along with their eminent antimicrobial activity against planktonic microbes, it was proved that EOs can demonstrate an antibacterial activity against biofilm-embedded microorganisms [16,17,18,19]. The phytochemical constituents of essential oils impede the process of microbial resistance since they are distinguished by exceptional combinations capable of inhibiting the processes of biofilm progression, such as phenols [20] and sulfur-containing compounds [21].

Biofilm control by EO application has recently increased to preclude biofilm production on foods and environmental surfaces [22,23,24]. Garlic and thyme essential oils, in particular, are regarded as natural antimicrobial sources in the food sector, in addition to preservation and flavoring ingredients [25,26,27,28]. Garlic (*Allium sativum*) essential oil (GEO) is a member of the Alliaceae family, and its biological effects are associated with sulfur-containing compounds such as allicin and other functionally active components [29,30]. Furthermore, a variety of aromatic bioactive components, including thymol and carvacrol, that play essential roles as antibacterial and antioxidative agents, were found in thyme (*Thymus vulgaris*) essential oil (TEO) [31].

Despite the importance of meat-producing animals and slaughter practices, few studies have been conducted on *S. typhimurium* in beef carcasses and the surfaces of the processing environment at slaughterhouses. Furthermore, various studies on the antimicrobial capabilities of plant compounds against *S*. *typhimurium* have been documented; nevertheless, their antibiofilm effects are far less well established [32,33,34,35]. To the best of the authors` knowledge, there are no previous studies that investigated the antibiotic-resistant and biofilm-producing *S. typhimurium* isolated from abattoir samples, along with the anti-biofilm impact of traditional oils on these bacterial strains. Hence, this study aimed at investigating the anti-biofilm effects of garlic and thyme essential oils against *S*. *typhimurium* at different temperatures (4 °C and 37 °C), as well as the virulence genes (*InvA, SdiA* and *Stn*) and the antimicrobial susceptibility profile of *S*. *typhimurium*.

## 2. Results

### 2.1. Prevalence of Salmonella Species in the Examined Abattoir Samples

*Salmonella* species were observed in abattoir samples with different percentages ranging from 25% in wall samples to 30% in abattoir effluents and floor samples. However, *Salmonella* failed to be isolated from carcass surfaces (Table 1). The serological identification of *Salmonella* species declared that *S. typhimurium, S. enteritidis, S. tsevie, S. montevideo, S. infantis, S. shubra, S. shangani*, and *S. tamale* were detected in five (5%), three (3%), three (3%), two (2%), one (1%), one (1%), one (1%), and one (1%) of the examined abattoir samples, respectively.

### 2.2. Virulence Gene Detection and Antibiotic Susceptibility of S. typhimurium Isolates

**Figure 1:**
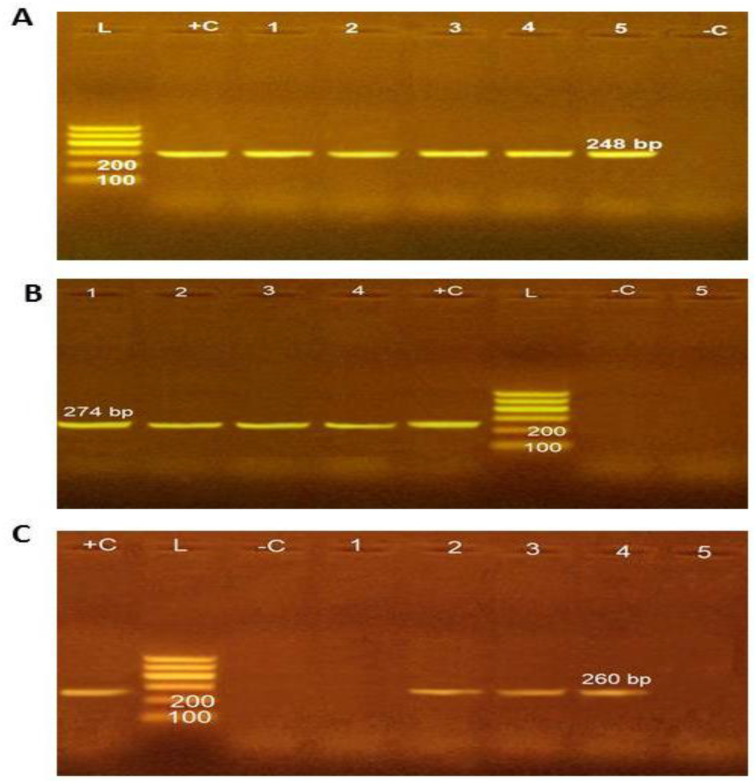
Molecular characterization of *S. typhimurium* virulence genes. (**A**) *Salmonella typhimurium*- *InvA* gene in 1.5% agarose gel (L: 100 bp ladder; −C: negative control; +C: positive control, from 1 to 5: *Salmonella* positive isolates for *InvA* gene). (**B**) *Salmonella typhimurium SdiA* gene in 1.5% agarose gel (L: 100 bp ladder; −C: negative control; +C: positive control; 1.2.3.4 *Salmonella* positive isolates; 5: negative *Salmonella* for *SdiA*). (**C**) *Salmonella typhimurium Stn* gene in 1.5% agarose gel (L: 100 bp ladder; −C: negative control; +C: positive control; 2.3.4 *Salmonella* positive isolates; 1,5: negative *Salmonella* for *Stn*). (Positive control: ATCC 43971, Negative control: PCR mixture without DNA template).

Figure 1 shows the virulence genes investigated in the five identified *S*. *typhimurium* isolates (two from abattoir effluents, two from walls, one from floors). The obtained results revealed that all five isolates were positive for the *InvA* gene; nevertheless, the *SdiA* gene was observed in four isolates (two from abattoir effluents, one from walls, one from floors). Regarding *Stn*, three isolates (one from abattoir effluents, one from walls, one from floors) were recorded as positive.

In the present work, Table 2 depicts the susceptibility of five *S. typhimurium* isolates to 16 antimicrobial agents. Obviously, the resistance of *S. typhimurium* isolates against erythromycin and oxacillin was 100% and 80%, respectively. However, 80% of *S. typhimurium* isolates demonstrated susceptibility to ciprofloxacin, amoxycillin, gentamicin, and ipipenem. Moreover, the susceptibility to nalidixic acid, sulphamethoxazol, amikacin, kanamycin, ampicillin, cefotaxime, meropenem, and cefazolin was 60%. Furthermore, the minimum effectiveness was revealed by tetracycline (20%) and clindamycin (40%). The resistance profile of each *S. typhimurium* isolate is presented in Table 2. All isolates were categorized as multi-drug resistant (MDR) *S. typhimurium*, and their multiple antibiotic resistance (MAR) index ranged from 0.125 to 1.00, with an average of 0.49.

### 2.3. Chemical Composition and Antibiofilm Activity of EOS

Table 3 shows the chemical composition analysis of the tested EOs, illustrating the main constituents obtained by GC–MS analysis. In this work, the EO resulting from *Allium sativum* was chiefly composed of allyl trisulfide (36.35%), followed by diallyl disulfide (21.55%), 2-Vinyl-4H-1,3-dithiine (7.77%), and allyl methyl trisulfide (7.34%), while the main components of EO obtained from *Thymus vulgaris* contained p-cymene (34.25%), thymol (28.86%), carvacrol (5.52%), and anethole (4.51%).

The biofilm-forming capability of *S. typhimurium* isolates and the antibiofilm effect of EOs (GEO and TEO) are presented in Table 4, Table 5 and Table 6. At 4 °C, the isolates were categorized as non-producers (three isolates) and moderate producers (two isolates), whereas at 37 °C, they were identified as non-producer (one isolate), weak (one isolate), moderate (two isolates), and strong (one isolate). The MTP assay results revealed that the EO of *Allium sativum* (GEO) inhibited *S*. *typhimurium* biofilm production at both temperatures in MIC/2 (1/128μL/mL), MIC/4 (1/256μL/mL), and MIC/8 (1/512μL/mL) concentrations. Regarding the EO of *Thymus vulgaris* (TEO), the different sub-MICs used, MIC/2 = 1/64 μL/mL, MIC/4 = 1/128 μL/mL, and MIC/8 = 1/256 μL/mL only reduced the degree of biofilm produced by the isolates.

## 3. Discussion

### 3.1. Prevalence of SalmonellaSpecies in the Examined Abattoir Samples

In this study, the *Salmonella* isolation rate of the examined abattoir floors (30%) was greater than those informed in previous works, including 13.33% in Sokoto abattoir, Nigeria [36]; 18% in Hawassa, Southern Ethiopia [37]; and 6.3% in Abuja abattoirs, Nigeria [38]. Furthermore, *Salmonella* species were recovered from 25% of abattoir walls, which is comparable to El-Gohary et al. [39], who recovered *Salmonella* isolates from abattoir walls with a prevalence of 30%. In contrast, some studies revealed that *Salmonella* could not be detected from abattoir walls [40].

*Salmonella* species failed to be isolated from carcass swabs in the current study. These discoveries are in agreement with the previous findings reported by Kikuvi et al. [41], since *Salmonella* could not be detected from beef surfaces slaughtered in Kenya slaughterhouses. On the other hand, Saad et al. [42] observed *S*. *enteritidis* and *S*. *typhimurium* in 4% of the investigated beef surfaces. However, Nouichi and Hamdi [1] isolated *Salmonella* species from brisket and rump with a contamination rate of 33.33% and 8.33%, respectively. Additionally, Chong et al. [43] recovered *Salmonella* from brisket and rump with a prevalence of 10%. The variation of isolation level, even absence or presence, might be attributed to the introduction of healthy or *Salmonella* carrier cattle to slaughter [44]. The prevalence of *Salmonella* in abattoir effluents was consistent with those obtained in Ogbete, Nigeria [45], and in Afikpo, South Eastern Nigeria [46]. The abattoir floors were the most contaminated samples, similar to the findings of Abdalla et al. [47] in Sudan. Microorganisms, such as *Salmonella*, carried by the animals on their skin and excrement, as well as blood droppings and viscera rupture, can cause high floor contamination [48,49]. Since a wide range of slaughter practices are conducted on the floor, extra precautions are needed to eliminate carcass contamination.

### 3.2. Molecular Characterization of S. typhimurium Virulence Genes

In the current work, the high PCR detection rate of the *InvA* (100%), *SdiA* (80%), and *Stn* (60%) genes suggests that these genes are essential virulence genes for *S. enterica* serotypes to convey their pathogenicity in the host [50]. The *InvA* gene is required for full *Salmonella* virulence as it enhances the internalization required for deeper tissue invasion [51]. Similarly, Rabins et al. [52] and Zadernowska and Chajęcka-Wierzchowska [53] discovered the *InvA* gene in all *S*. *typhimurium* isolated from beef samples. Moreover, *Stn* is a virulence gene responsible for *Salmonella* enterotoxicity [54]. Our results were comparable to Thung et al. [55], who detected the *Stn* gene in 50% of *S. typhimurium* isolates recovered from beef samples [55]. Furthermore, the *SdiA* gene is involved in epithelial cell adhesion and biofilm development via quorum sensing [56,57,58,59]. The results obtained are consistent with Yin et al. [60], who detected the *SdiA* gene in >75% of biofilm-producing *S. typhimurium* isolates. Additionally, Turki et al. [61] recovered the *SdiA* gene from all investigated *S. typhimurium* isolates.

### 3.3. Antibiotic Susceptibility of Salmonella typhimurium Isolates

The rising pattern of multi-resistance in *S. typhimurium* resulted from the misuse and overuse of antibiotics [62,63]. The current findings are comparable to those obtained by Abd El Tawab et al. [64], who demonstrated that *S. typhimurium* was highly susceptible to gentamycin (85%) and ciprofloxacin (80%), but resistant to erythromycin (40%). Similarly, Gebremedhin et al. [65] discovered that all tested *S. typhimurium* were highly sensitive to cefotaxime (85%), gentamycin (80%), amikacin (75%), and ciprofloxacin (60%). Furthermore, Gutema et al. [66] and Jaja et al. [67] found that the resistance pattern of *S*. *typhimurium* in beef samples was 100% for ampicillin and sulphamethoxazole in Ethiopia and South Africa, respectively. The high sensitivity of *S*. *typhimurium* to amikacin and ciprofloxacin in our study could be attributed to the limited use of these medications due to their high cost and difficulty to be purchased without a prescription in private pharmacies [68]. The MAR index value of 0.2 or above indicated contamination from high-hazard sources, putting consumers at risk [69].

### 3.4. Salmonella Typhimurium Biofilm Formation

The obtained results revealed that the incubation temperature significantly impacted *S*. *typhimurium’s* ability to generate biofilm. Due to its effect on bacterial migration toward the biofilm surface, the temperature has a negative impact on biofilm production [70]. In addition to the obtained results, Yin et al. [60] isolated one *S. typhimurium* from beef samples and classified it as a moderate biofilm producer at 37 °C but found it to be a non-biofilm producer at 4 °C. Moreover, Borges et al. [71] evaluated eight *S*. *typhimurium* isolates obtained from beef samples and classified them into seven weak biofilm and one medium biofilm producers at 37 °C, while the isolates were classified as three non-biofilm and five weak biofilm producers at 4 °C. The observed differences in biofilm formation between the previously mentioned studies could be thanks to many factors such as strain variation, incubation time, media, and temperature [72]. The findings revealed an association between the existence of the *SdiA* gene in *S*. *typhimurium* and the ability to form biofilms (Table 6). This relationship was supported by the findings of Wang et al. [73] and Lamas et al. [74].

According to the obtained results in Table 6, five *S*. *typhimurium* isolates recovered from abattoir samples, one isolate produced strong biofilm and was resistant to all the tested antibiotics (16 antibiotics), whereas two isolates showed moderate biofilm production and exhibited resistance to 12 and 6 antibiotics. Additionally, the other two isolates were resistant to three (weak biofilm producer) and two antibiotics (non-biofilm producer) at 37 °C. Furthermore, at 4 °C, two isolates of *S. typhimurium* with a high MAR index (>0.5) produced moderate biofilms, whereas three isolates with a low MAR index (<0.5) produced no biofilms. These findings were consistent with those of Kim and Wei [75] and Donlan and Costerton [76], who discovered that strong biofilm formers were also multidrug-resistant bacteria. However, these results were different from those of Ghasemmahdi et al. [77] and Wang et al. [78], who assumed that the association between biofilm formation and antibiotic resistance in *S*. *typhimurium* isolates is weak. Additionally, antibiotic resistance may be linked to the membrane permeability and metabolic activity changes caused by the structural and physiological features of biofilm cells [79].

### 3.5. Chemical Compositions and Antibiofilm Activity of Garlic and Thyme Essential Oils

The outcomes of the chemical composition of the EOs are similar to those of Zhang et al. [80] and Somrani et al. [81], who reported similar significant compounds while analyzing their GEO to those informed in our study. Our findings differed from those obtained by Alni et al. [63], who identified diallyl tetrasulfide, allyl disulfide, nitrosothymol, and 1H-1,2,4-triazole, 3-thiol-5-methyl as the main components of GEO in Iran. This difference might be attributed to genetic diversity, geographical origin, harvesting factors, or even extraction procedures [82]. With regard to the TEO, the major components were p-cymene (34.25%), thymol (28.86%), carvacrol (5.52%), and anethole (4.51%), suggesting that the EO studied belongs to the p-cymene chemotype, as previously described in Brazil [83] and Croatia [84]. The chemical composition of the TEO in this research is away from that previously found by Morocco [85] and Spain [86], where camphor and linalool were classified as the chemotypes of TEO.

Since there is a link between the chemical compositions of the most common oils and their antimicrobial properties [87], the biological effect of GEO is thought to be due to allyl sulfides (allyl methyl trisulfide, diallyl disulfide, and Diallyl tetrasulfide), which inhibit bacterial growth by interacting with cellular protein sulphydryl groups [88,89]. Moreover, it is evident that the antimicrobial activity of TEO is related to p-cymene and thymol (Table 3). The antibiofilm properties of p-cymene are mediated by a change in the plasma membrane lipid, which disrupts cytoplasmic membrane permeability and fluidity [90,91]. Analogous outcomes were informed by Alni et al. [63], who demonstrated the effectiveness of essential garlic oil with different concentrations of MIC 0.5-0.98 μL/mL in inhibiting all biofilm produced by *S. typhimurium*. Additionally, El-Azzouny et al. [92] elucidated the higher antibacterial activity of GEO against all *S. typhimurium* isolates with an MIC 0.125–1 μg/mL than TEO with an MIC 0.5–8 μg/mL, suggesting that GEO has a greater antibacterial effect against Gram-negative bacteria (*Salmonella* spp.) than TEO. Miladi et al. [93] demonstrated that TEO inhibited only 50% of biofilm formation by the recovered *S. typhimurium* isolates at an MIC extending from 0.106 to 0.725 mg/mL, and they attributed this activity to its phenolic components, particularly thymol [94]. Furthermore, Guillín et al. [95] revealed that TEO had an antibiofilm effect on *S. typhimurium* with an MIC 0.37 ± 0.7 μg/mL and an inhibition percentage higher than 60%. In contrast to our results, Axmann et al. [96] investigated the more significant effect of thyme oil than garlic oil on preventing *S. typhimurium* biofilm and found that a sub-MIC with 80 ppm thyme oil reduced the developed biofilm, while garlic oil had no effect. The differences between our findings and those of other researchers are most likely attributable to the plant diversities, locations, harvesting periods, and extraction methods [82,85].

## 4. Materials and Methods

### 4.1. Samples Collection

One hundred swab samples were collected between March and August 2021 from two sources namely, specific parts of beef carcasses (brisket and rump surfaces, 20 of each) and surfaces of the processing environment (abattoir effluents, floors, and walls, 20 of each) at Abu Hammad city abattoir, Sharkia Governorate, Egypt. Carcass samples were collected following de-hiding and post-evisceration. The obtained swabs were suspended in 25 mL of peptone water 1% (Oxiod, CM9).

### 4.2. Salmonella Isolation and Identification

The pre-enrichment of *Salmonella* was performed by incubating the original suspension containing the swabs at temperature of 37 °C for a period of 24 h. Next, 1 mL of pre-enriched peptone water was enriched in Rappaport Vassiliadis broth at temperature of 42 °C for a period of 24 h. A loopful was streaked on XLD agar, incubated at temperature of 37 °C for a period of 24 h, and red colonies with a black center were selected [97]. Five *S. typhimurium* isolates were subjected to biochemical analysis [98], serological identification [99], and then genomic DNA was extracted utilizing the QIAamp DNA kit according to the producer’s instructions. Table 7 described the primer sequences for the identification of virulent genes. The target genes included *InvA* (*Salmonella* invasion gene), *SdiA* (quorum sensing gene), and *Stn* (*Salmonella* enterotoxin).

### 4.3. Antibiogram Analysis

The antibiotic sensitivity testing of *S. typhimurium* was performed using a single diffusion assay against 16 antibiotic discs of varying concentrations [103]. The following antimicrobial discs were used: erythromycin (E), oxacillin (OX), clindamycin (CL), tetracycline (T), nalidixic acid (NA), sulphamethoxazol (SXT), amikacin (AK), kanamycin (K), ampicillin (AM), cefotaxime (CF), ciprofloxacin (CP), amoxicillin (AMX), gentamicin (G), meropenem (M), cefazolin (CZ), and ipipenem (IPM). Each strain was streaked on Mueller–Hinton agar (Himedia, Mumbai, India), and the drug-impregnated discs were placed on the agar medium surface. The multiple antibiotic resistance (MAR) index was detected.

### 4.4. MicrotiterPlate Assay (MTP) Investigation of Biofilm

The microtiter plate method (MTP) was utilized to assess the biofilm production of *S*. *typhimurium* isolates, as previously explained by Nair et al. [104], with some modifications. In brief, 180 μL of brain heart infusion (BHI) broth were filled into polystyrene microplate wells, and 20 μL of each isolate’s culture cultivated in BHI were distributed in triplicate into the wells of the microplate. In the negative control wells, which were done in triplicate, only 200 μL of broth was used. After inoculating the plates and incubating them aerobically at 37 °C for 24 h, the contents of the plates were drained, and the wells were washed correctly three times with phosphate buffer saline (PBS) (pH 7.2). Subsequently, 200 μL of methanol were added to each well to fix the adherent cells. For 10 min, the attached bacterial cells were stained with 200 μL of 0.5 percent (*w*/*v*) crystal violet (Fluka, Gillingham, UK). After staining, the plates were washed three times with PBS and air-dried before resolving with 250 μL 33% glacial acetic acid. After the negative control was adjusted to zero, the optical density (OD) of the stained attached bacteria was determined at 570 nm utilizing a microplate reader. This experiment was conducted in triplicates, and the data is expressed as an average with a standard deviation. The strains were grouped as non, weak, moderate, or strong biofilm makers following the equations developed by Stepanović et al. [105].

### 4.5. Gas Chromatography/Mass Spectrometry (GC/MS) Analysis of EOs

The essential oils of garlic (*Allium sativum*) and thyme (*Thymus vulgaris*) were prepared in 95% (*v*/*v*) ethanol and kept at 4°C for further experiments. The chemical composition of EOs was determined using a GC–MS system (6890 N gas chromatograph; Agilent Technologies Inc., Palo Alto, CA, USA) fitted with an HP-5MS capillary column (30 m × 0.25 mm × 0.25 µm). The temperature of the oven was adjusted as follows: 50 °C (initial), then 5 °C /min to 220 °C, and finally 10 °C /min to 280 °C and held for 5 min. The transfer line temperature was 250 °C. EO samples of 1 µL were injected in the split mode at a ratio of 1:20. Helium was used as the carrier gas with a 1 mL/min flow rate.

### 4.6. Measuring the Minimal Inhibitory Concentration (MIC) of the Investigated EOs

The microdilution broth scheme, as formerly used by Duarte et al. [106], was used to determine the minimum inhibitory concentration (MIC) of the essential oils with some modifications. In a 96-well microtiter plate containing Müeller–Hinton broth (MHB), two-fold serial dilutions of essential oil ranging from 0.5 µL/mL to 0.004 µL/mL were adequately prepared, and 1% Dimethyl sulfoxide (DMSO) was added to enhance oil solubilization. The isolates were enriched on brain heart infusion agar media (BHI), incubated at temperature of 37 °C for a period of 24 h, adjusted to a turbidity of 0.5 McFarland (1.5 × $10^{8}$ CFU/mL), and applied to each well. Under aerobic conditions, the microtiter plates were maintained at the incubator for 24 h at 37 °C. Turbidity was employed to detect visible bacterial growth on the plates. The MIC of an EO was described as the fewest concentration of the EO that generated no visible growth.

### 4.7. Anti-Biofilm Efficiency of Essential Oils versus S. typhimurium Isolates

The MTP assay was utilized to measure the inhibitory and disruptive impacts of the examined essential oils on *S*. *typhimurium* biofilms, as performed by Sharifi et al. [107]. Briefly, the sterile 96-well polystyrene plates were loaded with 100 μL of BHI containing the EO concentrations MIC/2, MIC/4, and MIC/8. Afterward, 100 μL of the bacterial culture with a concentration of 1.5 × $10^{8}$ CFU/mL were added to each well. The bacteria with BHI medium were used as a positive control. The mixture of DMSO and BHI was used as a negative control. After incubation, the plates were triple rinsed with physiological saline (200 μL /well) in order to eliminate the weakly adhered and air-dried cells. Following that, the attached cells of the biofilm were fixed for 15 min with 200 μL of methanol. After removing the methanol, 200 μL of a 0.1% Safranin dye was provided for 15 min. The plates were flushed with physiological saline and air-dried again, 100 μL of ethanol (96 percent) was added to each well, and shaken for 5 min. The optical densities (ODs) were estimated at 490 nm using an Elisa reader.

### 4.8. Statistical Analysis

Biofilm formation by *S. typhimurium* and the antibiofilm activity of EOs were determined at 4 °C and 37 °C. The trials in this study were conducted in triplicate. The degree of biofilm production by *S. typhimurium* strains was represented as strong, moderate, weak, and non-biofilm producer. ANOVA test was used to determine the differences in biofilm formation at the two temperatures. The statistical analyses were applied utilizing SPSS software version 25. Data are summarized as the average ± SD, and significant differences were considered at a significant level of *p* ˂ 0.05.

## 5. Conclusions

*Salmonella* species were detected with varying percentages in the examined abattoir samples, with *S. typhimurium* being the most prevalent isolate. Furthermore, the relationship between the high MAR index and the propensity of *S*. *typhimurium* isolates to form biofilm at chilling temperature (3 ± 1 °C) indicates a substantial source of carcass contamination inside the abattoir chilling rooms. The present study reported that the EOs of garlic and thyme had a strong impact against *S*. *typhimurium* in vitro at various sub-minimum inhibitory concentrations, particularly garlic essential oil (GEO). Consequently, natural EOs may be a potential natural antibacterial option for food processing facilities. Considering their multi-component composition, EOs prevent the emergence of bacterial resistance compared to conventional antibiotics. The outcomes of the present work could provide beneficial details for the bioactivity of GEO and TEO in controlling antibiotic-resistant and biofilm-producing *S. typhimurium*. Therefore, the information extracted from this study could be utilized in different food preservatives and pharmaceutical applications.

## Figures and Tables

**Table 1:** Prevalence of *Salmonella* species in the examined abattoir samples.

Serotypes	Samples (20 for Each)					Total
	Rump	Brisket	Abattoir Effluents	Floors	Walls	
*S. typhimurium*	-	-	2 (10%)	1 (5%)	2 (10%)	5 (5%)
*S*. *enteritidis*	-	-	1 (5%)	2 (10%)	-	3 (3%)
*S*. *tsevie*	-	-	-	1 (5%)	2 (10%)	3 (3%)
*S*. *montevideo*	-	-	1 (5%)	1 (5%)	-	2 (2%)
*S*. *infantis*	-	-	1 (5%)	-	-	1 (1%)
*S. shubra*	-	-	1 (5%)	-	-	1 (1%)
*S*. *shangani*	-	-	-	1 (5%)	-	1 (1%)
*S*. *tamale*	-	-	-	-	1 (5%)	1 (1%)

**Table 2:** Antimicrobial susceptibility and resistance profile of *S. typhimurium*.

Antimicrobial Agents		Concentration (μg/Disc)	Sensitive		Intermediate		Resistant	
			Number	%	Number	%	Number	%
Erythromycin (E)		15	-	-	-	-	5	100
Oxacillin (OX)		1	-	-	1	20	4	80
Clindamycin (CL)		10	2	40	-	-	3	60
Tetracycline (T)		30	1	20	1	20	3	60
Nalidixic acid (NA)		30	3	60	-	-	2	40
Sulphamethoxazol (SXT)		25	3	60	-	-	2	40
Amikacin (AK)		30	3	60	-	-	2	40
Kanamycin (K)		30	3	60	1	20	1	20
Ampicillin (AM)		10	3	60	1	20	1	20
Cefotaxime (CF)		30	3	60	1	20	1	20
Ciprofloxacin (CP)		5	4	80	-	-	1	20
Amoxycillin (AMX)		30	4	80	-	-	1	20
Gentamicin (G)		10	4	80	-	-	1	20
Meropenem (M)		10	3	60	1	20	1	20
Cefazolin (CZ)		30	3	60	-	-	2	40
Ipipenem (IPM)		10	4	80	-	-	1	20
**Isolates**		**Resistance profile**			**Number of antibiotics**		**Number isolates (%)**	**MAR index**
**Source**	**Pattern**							
Abattoir effluents	I	E, OX, CL, T, NA, SXT, AK, K, AM, CF, CP, AMX, G, M, CZ, IPM			16		1 (20%)	1
Walls	II	E, OX, CL, T, NA, SXT, AK, K, AM, CF, CP, AMX			12		1 (20%)	0.750
Floors	III	E, OX, CL, T, NA, SXT			6		1 (20%)	0.375
Abattoir effluents	IV	E, OX, CL			3		1 (20%)	0.188
Walls	V	E, OX			2		1 (20%)	0.125
**Average**		**0.49**						

MAR refers to multiple antibiotic resistance index (a/b), where a and b denote the antibiotics number to which the isolates are resistant and the sum of tested antibiotics (16), respectively.

**Table 3:** Chemical compositions of the *Allium sativum* and *Thymus vulgaris* essential oils.

No	*Allium sativum*			*Thymus vulgaris*		
	Compound	Content * (%)	RT	Compound	Content * (%)	RT
1	1,2-Dithiolane	0.57	3.156	Propyl acetate	1.60	3.923
2	Diallyl sulfide	0.49	3.391	α-Pinene	2.70	6.389
3	Allyl methyl disulfide	1.76	4.661	Camphene	1.19	6.755
4	1,2-Dithiacyclopentene	1.44	5.805	β-Pinene	1.95	7.436
5	Diallyl disulfide	21.55	10.217	β-Myrcene	0.93	7.768
6	Allyl (E)-1-Propenyl disulfide	0.88	10.623	p-Cymene	34.25	8.741
7	Allyl (Z)-1-Propenyl disulfide	3.47	10.858	D-Limonene	1.02	8.815
8	Allyl methyl trisulfide	7.34	12.002	Eucalyptol	1.95	8.884
9	3-Vinyl-1,2-dithi-4-ene	2.95	13.513	γ-Terpinene	1.41	9.610
10	4H-1,2,3-Trithiine	4.79	13.719	Linalool	3.10	10.715
11	2-Vinyl-4H-1,3-dithiine	7.77	14.343	Camphor	1.61	12.620
12	Allyl trisulfide	36.35	16.958	Terpinene-4-ol	1.45	12.935
13	Allyl propyl trisulfide	0.71	17.249	Methyl thymol	2.80	14.497
14	5-Methyl-1,2,3,4-tetrathiane	1.44	18.508	Methyl carvacrol	1.74	14.754
15	1,2-Dithiole	1.78	19.584	Anethole	4.51	15.905
16	Diallyl tetrasulfide	2.40	22.960	Thymol	28.86	16.065
17	Octathiocane	3.10	32.951	Carvacrol	5.52	16.294
18	3,11a-Dimethylhexadecahydro-3H-naphtho [2′,1′:4,5] indenol[1,7a–c] furan-1-one	1.21	46.226	β-Caryophyllene	2.11	19.498
19	-			Caryophyllene oxide	1.30	23.492
Total		100%		Total	100%	

* The chemical contents were noticed from a peak area proportionate to the total peak area in GC–MS analysis. RT = Retention Time.

**Table 4:** Development of biofilm by *S*. *typhimurium* in untreated and treated groups at 4 °C.

Groups	Degree of Biofilm Production	Control (Untreated Group)	GEO-Treated Group			TEO-Treated Group		
			MIC/2	MIC/4	MIC/8	MIC/2	MIC/4	MIC/8
Non-producer No (%) Average OD ± SD	-	3(60%) 0.048 ± 0.002	2(40%) 0.075 ± 0.01	2(40%) 0.080 ± 0.01	2(40%) 0.083 ± 0.02	-	-	-
Biofilm producer No (%) Average OD ± SD	Weak	-	-	-	-	2(40%) 0.473 ± 0.05	2(40%) 0.485 ± 0.06	2(40%) 0.496 ± 0.06
	Moderate	2(40%) 0.24 5 ± 0.008	-	-	-	-	-	-
	Strong	-	-	-	-	-	-	-
Overall biofilm producers at 4 °C/24 h incubation		2(40%)	-	-	-	2(40%)	2(40%)	2(40%)

GEO: Garlic essential oil. TEO: Thyme essential oil.SD: Standard deviation. OD: Optical density. MIC of GEO = 1/64. MIC of TEO = 1/32. Regarding GEO concentrations: MIC/2 = 1/128 μL/mL, MIC/4 = 1/256 μL/mL, MIC/8 = 1/512 μL/mL. Regarding TEO concentrations: MIC/2 = 1/64 μL/mL, MIC/4 = 1/128 μL/mL, MIC/8 = 1/256 μL/mL.

**Table 5:** Development of biofilm by *S. typhimurium* in untreated and treated groups at 37 °C.

Groups	Degree of Biofilm Production	Control (Untreated Group)	GEO-Treated Group			TEO-Treated Group		
			MIC/2	MIC/4	MIC/8	MIC/2	MIC/4	MIC/8
Non-producer No (%) Average OD ± SD	-	1(20%) 0.06 ± 0.007	4(80%) 0.055 ± 0.001	4(80%) 0.065 ± 0.01	4(80%) 0.074 ± 0.01	1(20%) 0.411 ± 0.61	1(20%) 0.428 ± 0.61	1(20%) 0.437 ± 0.61
Biofilm producer No (%) Average OD ± SD	Weak	1(20%) 0.12 ± 0.035	-	-	-	3(60%) 0.732 ± 0.09	3(60%) 0.743 ± 0.09	3(60%) 0.748 ± 0.09
	Moderate	2(40%) 0.246 ± 0.013	-	-	-	-	-	-
	Strong	1(20%) 0.311 ± 0.034	-	-	-	-	-	-
Overall biofilm producers at 37 °C/24 h incubation		4(80%)	-	-	-	3(60%)	3(60%)	3(60%)

GEO: Garlic essential oil. TEO: Thyme essential oil.SD: Standard deviation. OD: Optical density. MIC of GEO = 1/64. MIC of TEO = 1/32. GEO concentrations: MIC/2 = 1/128 μL/mL, MIC/4 = 1/256 μL/mL, MIC/8 = 1/512 μL/mL. Regarding TEO concentrations: MIC/2 = 1/64 μL/mL, MIC/4 = 1/128 μL/mL, MIC/8 = 1/256 μL/mL.

**Table 6:** Virulence genes, antibiotic resistance, and biofilm-forming capability of *S. typhimurium* isolates.

Isolates		Virulence Genes			Antibiotic Resistance (No)	Degree of Biofilm Formation					
						Control (Untreated)		GEO		TEO	
No.	Source	*InvA*	*SdiA*	*Stn*		4 °C	37 °C	4 °C	37 °C	4 °C	37 °C
1	Abattoir effluents	+	+	−	16	Moderate	Strong	Non-producer	Non-producer	Weak	Weak
2	Walls	+	+	+	12	Moderate	Moderate	Non-producer	Non-producer	Weak	Weak
3	Floors	+	+	+	6	Non-producer	Moderate	-	Non-producer	-	Weak
4	Abattoir effluents	+	+	+	3	Non-producer	weak	-	Non-producer	-	Non-producer
5	Walls	+	−	−	2	Non-producer	Non-producer	-	-	-	-

**Table 7:** Oligonucleotide primers for PCR reactions used for the amplification of the target genes in *S. typhimurium* isolates.

Primer	Oligonucleotide Sequence (5′ → 3′)	Product Size (bp)	Annealing (°C)	References
*SdiA*	AATATCGCTTCGTACCAC	274	49	[100]
	GTAGGTAAACGAGGAGCAG			
*Stn*	CTTTGGTCG TAA AATAAGGCG	260	54	[101]
	TGCCCAAAGCAGAGAGATTC			
*InvA*	GTGAAATTATCGCCACGTTCGGGCAA	248	55	[102]
	TCATCGCACCGTCAAAGGAACC			

## Data Availability

Data of this work is included in the main document.

## Sample Availability

Samples of the compounds are available from the authors.
